# Five glutathione *S*-transferase isozymes played crucial role in the detoxification of aflatoxin B_1_ in chicken liver

**DOI:** 10.1186/s40104-025-01189-7

**Published:** 2025-04-08

**Authors:** Jiang Deng, Zhe Peng, Zhiyuan Xia, Yixin Mo, Lijia Guo, Jintao Wei, Lvhui Sun, Meng Liu

**Affiliations:** 1 State Key Laboratory of Agricultural Microbiology, Hubei Hongshan Laboratory, Frontiers Science Center for Animal Breeding and Sustainable Production, Key Laboratory of Smart Farming Technology for Agricultural Animals of Ministry of Agriculture and Rural Affairs, College of Animal Sciences and Technology, Huazhong Agricultural University, Wuhan, Hubei 430070 China; 2 Hebei Panshuo Biotechnology Co., Ltd., Baoding, Hebei 071500 China; 3 Institute of Animal Husbandry and Veterinary Sciences, Hubei Academy of Agricultural Sciences, Wuhan, 430064 China

**Keywords:** Aflatoxin B_1_, Chicken, Detoxification, Enzymatic characteristics, GST isozymes

## Abstract

**Background:**

AFB_1_-8,9-exo-epoxide (AFBO) is the highly toxic product of Aflatoxin B_1_ (AFB_1_). Glutathione *S*-transferases (GSTs) play pivotal roles in detoxifying AFB_1_ by catalyzing the conjugation of AFBO with glutathione (GSH). Although there are over 20 GST isozymes that have been identified in chicken, GST isozymes involved in the detoxification process of AFB_1_ have not been identified yet. The objective of this study was to determine which GST isozymes played key role in detoxification of AFB_1_.

**Results:**

A total of 17 pcDNA3.1(+)-GST isozyme plasmids were constructed and the GST isozyme genes were overexpressed by 80–2,500,000 folds in the chicken Leghorn male hepatoma (LMH) cells. Compared to the AFB_1_ treatment, overexpression of GSTA2X, GSTA3, GSTT1L, GSTZ1-1, and GSTZ1-2 increased the cell viability by 6.5%–17.0% in LMH cells. Moreover, overexpression of five GST isozymes reduced the release of lactate dehydrogenase and reactive oxygen species by 8.8%–64.4%, and 57.2%–77.6%, respectively, as well as enhanced the production AFBO-GSH by 15.8%–19.6%, thus mitigating DNA damage induced by AFB_1_. After comprehensive evaluation of various indicators, GSTA2X displayed the best detoxification effects against AFB_1_. GSTA2X was expressed in *Pichia pastoris* X-33 and its enzymatic properties for catalyzing the conjugation of AFBO with GSH showed that the optimum temperature and pH were 20–25 °C and 7.6–8.6 as well as the enzymatic kinetic parameter *V*_*max*_ was 0.23 nmol/min/mg and the Michaelis constant was 86.05 μmol/L with the AFB_1_ as substrate.

**Conclusions:**

In conclusion, GSTA2X, GSTA3, GSTT1L, GSTZ1-1, and GSTZ1-2 played key roles in AFB_1_ detoxification, which will provide new remediation strategies to prevent aflatoxicosis in chickens.

**Supplementary Information:**

The online version contains supplementary material available at 10.1186/s40104-025-01189-7.

## Introduction

Aflatoxin B_1_ (AFB_1_) is a secondary metabolite produced by *Aspergillus flavus* and *Aspergillus parasiticus*, naturally occurring in a variety of human food and animal feed [1, 2]. Among the more than 20 identified aflatoxins, AFB_1_ is the most toxic and has been classified as Group I carcinogen by the International Agency for Research on Cancer (IARC), due to its hepatoxicity, nephrotoxicity, immunotoxicity, teratogenicity, and mutagenicity [3–6]. Climate change and global warming have exacerbated the risk of chronic aflatoxins exposure, affecting approximately five billion people worldwide, particularly in the developing countries [7–9]. Since its first identification in the ‘turkey X disease’, AFB_1_ has been a significant concern in food safety and public health [10]. Therefore, sufficiently understanding the adverse molecular effects of AFB_1_ on animals is crucial for developing effective remediation strategies to protect human and animal health from its threats [11–13].

The liver is the target organ and most important metabolic organ of AFB_1_ [14]. Ingestion of feed exposure to AFB_1_ exceeding the tolerance threshold leads to liver damage in chicken, including hepatocyte vacuolization, hemorrhagic necrosis, steatosis, cholestasis, and proliferation of bile duct epithelium [15–17]. After being absorbed into the liver, AFB_1_ is bioactivated into highly toxic AFB_1_-8,9-exo-epoxide (AFBO) and less toxic aflatoxin M_1_, Q_1_, and non-toxic P_1_ by hepatic microsomal cytochrome P450 (CYP450) [18–20]. In chicken, hepatic CYP1A1 and CYP2A6 are responsible for the predominant production of AFBO in the liver [18, 21]. It has been reported that AFBO, with highly activity and electrophilicity, can covalently bind to DNA and serum albumin lysine to form AFB_1_-N7-GUA and lysine adducts, resulting in DNA lesions, mutations and cytotoxicity [4, 6]. The detoxification pathway of AFB_1_ depends on the conjugation of AFBO with glutathione (GSH), which is catalyzed by glutathione *S*-transferase (GST) in liver [14]. GSTs are a diverse family of phase II detoxification enzymes, categorized into cytosolic, mitochondrial, and microsomal GSTs, with cytosolic GSTs [22]. Cytosolic GSTs, including alpha (GSTA), zeta (GSTZ), theta (GSTT), mu (GSTM), pi (GSTP), sigma (GSTS) and omega (GSTO) based on their chemical, physical and structural properties, play a dominant role in neutralizing electrophilic and carcinogenic substrates [22, 23]. Numerous studies have reported that different GST isozymes catalyzing the conjugation of AFBO with GSH vary among different species [9, 14]. For instance, GSTM1-1, GSTM2-2, GSTA1-1 and GSTA2-2 in humans, GSTM2-2 in *Macaca fascicularis*, GSTA3 in mice, GSTA5 in rats, GST and GST3 in duck, GSTA1, GSTA2, GSTA3, and GSTA4 in turkey play the primary role in the detoxification of AFBO [9, 14, 24, 25]. Currently, more than 20 GST isozymes have been identified in chickens; however, which GST isozymes are responsible for the detoxification of AFB_1_ in chicken liver remains unknown.

In the present study, we have cloned 17 GST isozymes and identified the key GST isozymes involved in detoxifying AFB_1_ in chicken liver through the determination of cell viability, lactate dehydrogenase (LDH) activity, production of reactive oxygen species (ROS) and AFBO-GSH, and DNA damage. GSTA2X was a crucial isoform and we further expressed it using *Pichia pastoris* X-33 and determined its enzymatic characteristics and kinetic parameters. Generally, this study aims to provide new regulatory targets for the development of nutritional strategies to mitigate AFB_1_-induced hepatotoxicity in chickens.

## Materials and methods

### Cloning and construction of GST isozymes overexpression plasmids

The total mRNA was extracted from the Cobb broiler to generate the cDNA using the reverse transcription kit (Takara Bio Inc., Kusatsu, Japan). Seventeen gene fragments of GST isozymes (*GSTALX1*, *GSTA2*, *GSTA2X*, *GSTAL2X*, *GSTA3*, *GSTAL3*, *GSTAL3X1*, *GSTA4*, *GSTA4LX1*, *GSTM2*, *GSTK1*, *GSTO2*, *GSTT1*, *GSTZ1X1*, *GSTZ1-1* and *GST1-2*) were obtained through polymerase chain reaction (PCR) using the relative primers (Additional file 1: Table S1) with homology arm. GST fragments with homologous arms were ligated through *Exnas II* (Vazyme Biotech Co., Ltd., Nanjing, China) into the linearization pcDNA3.1(+) digested by Hind III-HF (Vazyme Biotech Co., Ltd., Nanjing, China). The pcDNA3.1(+)-GST plasmids were then transformed into *E. coli* Top 10, and positive transformants were selected by ampicillin (25 mg/mL) resistance [26]. Then the PCR was carried out on the bacterial colonies using the universal primers (CMV-F: 5′-CGCAAATGGGCGGTAGGCGTG-3′; BGH: 5′-CAGGGTCAAGGAAGGCAC-3′) of the pcDNA3.1(+)-GST plasmids, followed by a final verification through sequencing (Tsingke Biotechnology Co., Ltd., Wuhan, China).

### Cell culture and transient transfection

The chicken Leghorn male hepatoma (LMH) cells were obtained from ATCC (Manassas, VA, USA). Cells were grown in DMEM/F12 supplemented with 10% fetal bovine serum, 100 μg/mL penicillin/streptomycin/gentamicin (Invitrogen, Gibco) and in the condition of 95% air and 5% CO_2_ humidified atmosphere at 37 °C. After the density of cells reached 70%−80%, pcDNA3.1(+)-GST plasmids were transfected using Lipo 2000 (Invitrogen) according to the manufacturer’s instructions. At 24 h post-transfection, the total RNA was isolated from LMH cells, and the relative mRNA abundance was quantified to assess transfection efficiency [27]. The target GST genes and their primers were listed in Additional file 2: Table S2.

### Cell viability, LDH content and intracellular ROS assays

After seeding the cells in the 96-well plate for 24 h post-transfection, they were treated with AFB_1_ at the concentration of 10, 25, 50, 100 and 200 μg/L. At 24 h post-treated, cell viability was determined and 30% inhibitory concentration (IC_30_) of AFB_1_ was calculated. Briefly, 10 μL of CCK-8 reagent (Biosharp, Hefei, China) was added to each well and then the 96-well plate was continuously incubated at 37 °C for 1 h. Then the absorbance at 450 nm wavelength was measured by a microplate reader (LabServ, Thermo Fisher Scientific, Waltham, MA, USA). At 24 h post-transfection with GST plasmids, cells were treated with AFB_1_ at the concentration of IC_30_ for 24 h. Then, the cell viability was determined, and 100 μL supernatant from cell culture medium was collected to measure LDH content using the specific kit (Nanjing Jiancheng Bioengineering Institute, China) according to the manufacturer’s protocol [28]. And the absorbance of each well was measured at 450 nm using a microplate reader. The ROS level was determined with the specific assay kits (S0033S; Beyotime Biotechnology, Shanghai, China). The equivalent number of cells were seeded in a 12-well plate and treated with AFB_1_ for 48 h. Cells were incubated with DCFH-DA at concentration of 10 μmol/L for 30 min at 37 °C. After three washes with free-serum media, cells were collected, counted, and analyzed for their fluorescence intensity using a fluorescence microplate reader (Thermo Fisher Scientific, Waltham, MA, USA) with its excitation and emission wavelengths at 488 nm and 525 nm [29, 30].

### Determination of AFBO-GSH concentration

The content of AFBO-GSH in the cell was measured as previously described with minor revision [9, 31]. Briefly, the cells and the medium were collected into 2-mL centrifuge tubes for vacuum freeze-drying after 24 h AFB_1_ (IC_30_) treatment. The freeze-dried samples were dissolved with 250 μL ice-cold methanol (Thermo Fisher Scientific, Waltham, MA, USA). This mixture was centrifuged at 15,000 r/min for 10 min at room temperature. The supernatant was then analyzed by reverse-phase HPLC (Agilent 1260 Infinity LC, USA) on a Welch XB-C18 column (5 μm, 250 mm × 4.6 mm) equipped with a fluorescence detector at the excitation and emission wavelengths of 365 and 440 nm, respectively [9]. The mobile phase was water/acetonitrile/methanol (60:20:20) (Thermo Fisher Scientific, Waltham, MA, USA) [9]. The quantification of AFBO-GSH was performed by HPLC peak area integration.

### Immunofluorescence staining analysis of γ-H2AX

Cells were grown on slides in 12-well plates and treated with AFB_1_ at 24 h post-transfection. After being treated for 24 h, cell slides were washed with cold PBS three times, and fixed with cold 4% paraformaldehyde for 10 min. Subsequently, cells were blocked with blocking buffer (PBS, containing 3% BSA, 0.3% Triton X-100, and 10% goat serum; purchased from Beyotime Biotechnology, Shanghai, China) for 30 min at room temperature. Then, cells were incubated with a 1:200 dilutions of primary anti-γ-H2AX antibody (ABclonal Technology, Wuhan, China) overnight at 4 °C, followed by three washes with PBS and incubation with FITC-labeled goat anti-rabbit IgG antibody for 1 h and mounted with DAPI [32]. Finally, cell slides were observed by a Leica DMi8 fluorescence microscope.

### Sequences alignment and phylogenetic analysis of GST proteins

The amino acid sequences of 17 GST proteins from *Gallus gallus* and 12 GST proteins which were vital in AFBO detoxification in another 5 different species (*Meleagris gallopavo*, *Homo sapiens*, *Mus musculus*, *Rattus norvegicus,* and *Macaca fascicularis*) were obtained from the NCBI Protein Database or Unified Protein Database [14]. All amino acid sequences of GSTs were aligned using Multiline (http://multalin.toulouse.inra.fr). Multiple alignments were performed with the full-length amino acid sequences of the GST class proteins using MEGA 11 (https://www.megasoftware.net/) [33], and the same software was used to construct a phylogenetic tree based on the Maximum-likelihood algorithm with bootstrap analysis of 1,000 iterations. Additionally, these protein domain sequences of these proteins were annotated by NCBI Batch CD-Search (https://www.ncbi.nlm.nih.gov/Structure/bwrpsb/bwrpsb.cgi) [34] and illustrator with TBtools [35]. Those with E-value < 0.0001 were selected.

### Construction of the GSTA2X expression plasmid and its expression in *P. pastoris* X-33

After a comprehensive comparison, the results showed that GSTA2X exhibited superior detoxification activity against AFB_1_ compared to the other four GST isozymes. The pcDNA3.1(+)-GSTA2X was used as a template to amplify the cDNA fragment encoding GSTA2X protein. The forward primer was (5′-GCGGCCGCCAGCTTTCTAGAatggctgggaaaccgaag-3′, the underlined was *XbaI* (Vazyme Biotech Co., Ltd., Nanjing, China) site), and the reverse primer was (5′-GAGATGAGTTTTTGTTCTAGAtta*ATGATGATGATGATGATG*gaaactgaatatttt-3′, the underlined was *XbaI* site, the italic was 6 × His tag). After purification, the GSTA2X fragment with homologous arms was ligated through *Exnas II* into the linearization pPICZαA digested by *XbaI*. Then, the recombinant plasmid pPICZαA-GSTA2X was transformed into *P. pastoris* X-33 by electroporation. Single colonies of the transformants were selected by PCR for expression [36]. After 72 h of methanol induction, the expressed extracellular GSTA2X protein samples were separated by 10% SDS-polyacrylamide gel electrophoresis (SDS-PAGE) and visualized by staining with Coomassie Brilliant Blue R-250 and silver staining reagent (Beyotime Biotechnology, Shanghai, China).

### Purification of recombinant GSTA2X and characterization of reGSTA2X

After 72 h-methanol induction, fermentation broth of re*P. pastoris* X-33 was centrifuged at 12,000 r/min at 4 °C for 10 min. The supernatant was mixed with Ni resin and incubated at 4 °C for 3 min on shaker. After that, the mixture was transferred into the column and pre-washed with wash buffer (PBS, contained 0, 10, or 30 mmol/L imidazole, pH = 7.6), followed by elution buffer (PBS, contained 300 mmol/L imidazole, pH = 7.6). The harvest protein was stored at −80 °C for subsequent analysis.

The activity of reGSTA2X in catalyzing the conversion of AFBO to AFBO-GSH were measured as described by Kim et al. [25]. Liver microsome from 6-week-old Sprague Dawley male rat (20 mg/mL, obtained from CHI Scientific, Inc., Massachusetts, USA) was used to generate AFBO; 150 mg/L AFB_1_ (diluted in dimethyl sulfoxide), 2 mmol/L NADPH, 5 mmol/L GSH, rat cytosol, and recombinant GSTA2X (2.5 μg total protein) were added at the same time. The mixture was incubated in AFBO trapping buffer (5 mmol/L MgCl_2_, 25 mmol/L KCl, 0.25 mmol/L sucrose, and 80 mmol/L potassium phosphate, pH = 7.6) to get a final volume of 200 μL. After incubation for 20 min with gentle shaking, the reactions were stopped by adding 200 μL cold methanol. Then the mixture was extracted overnight and centrifuged at 10,000 r/min and 4 °C to obtain the supernatant for HPLC analysis.

The optimal temperature and pH for the reGSTA2X was determined using the reaction system at different temperature from 15 to 60 °C and pH from 3.6 to 9.6. The thermal stability of reGSTA2X was determined by measuring the residual activity after the enzyme was incubated at 25 °C (or 30, 35, 40 °C) for 0, 30, 60, 90, 120 and 150 min. The group with the highest activity was set as control. To test the function mechanism of purified reGSTA2X under different conditions, it was incubated with the addition of divalent metal ions (Fe^2+^, Zn^2+^, Mn^2+^, Cu^2+^, and Mg^2+^) at the concentration of 1 mmol/L or 0.1% sodium dodecyl sulfonate (SDS). The changes in the reaction compared to the untreated control were measured. Kinetic constants of Michaelis constant (*K*_*m*_) and maximal reaction velocity (*V*_*max*_) were determined at pH 7.6 and 25 °C using the Lineweaver-Burk method [37].

### Data analysis

Data were analyzed using one-way ANOVA to determine the treatment effects by IBM SPSS Statistics software (version 21, IBM, USA). Data are presented as mean ± SEM. Duncan test and *P* value of < 0.05 were used to indicate statistical significance.

## Results

### Characterization of the detoxification ability of GST isozymes to AFB_1_

**Fig. 1:**
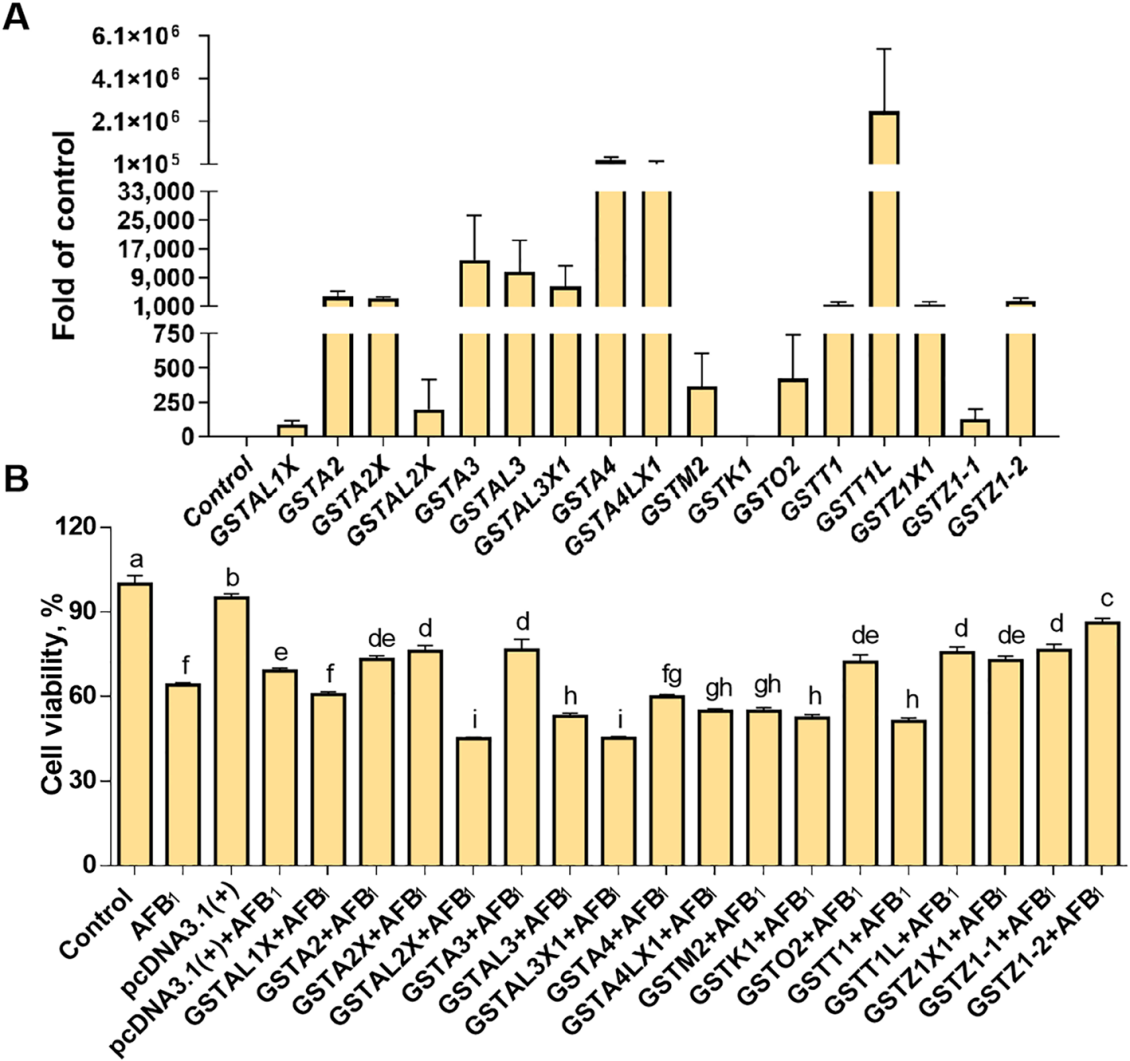
Transfected verification (**A**) and effects of overexpressed GST isozymes on viability (**B**) of LMH cell treated with AFB_1_. **A** Control, cells without any treatment; GSTs, the cells transfected with different pcDNA3.1(+)-GST plasmid. **B** Control, cells without any treatment; AFB_1_, cells treated with 100 μg/L AFB_1_; pcDNA3.1(+), the cells transfected with pcDNA3.1(+) plasmid; pcDNA3.1(+) +AFB_1_, the cells transfected with pcDNA3.1(+) plasmid plus AFB_1_ treatment; GSTs+AFB_1_, the cells transfected with different pcDNA3.1(+)-GSTs plus AFB_1_ treatment. Values are expressed as mean ± SD (*n* = 6). ^a–i^Different letters between groups represent significant differences, *P* < 0.05

Seventeen overexpressed GST vectors were constructed and transfected into LMH cells. Except for GSTK17 GST isozymes were upregulated by 80–2,500,000 folds than those in the control group (Fig. 1A). As shown in Fig. 1B, the overexpression of GSTZ2X, GSTA3, GSTT1L, GSTZ1-1, and GSTZ1-2 increased (*P* < 0.05) the cell viability by 6.9%, 7.4%, 6.5%, 7.3% and 17.0% in comparison to the group treated with pcDNA3.1(+) and AFB_1_.

### Mitigation effects of 5 GST isozymes on AFB_1_-induced elevation of LDH release, ROS and AFBO-GSH production and DNA damage

**Fig. 2:**
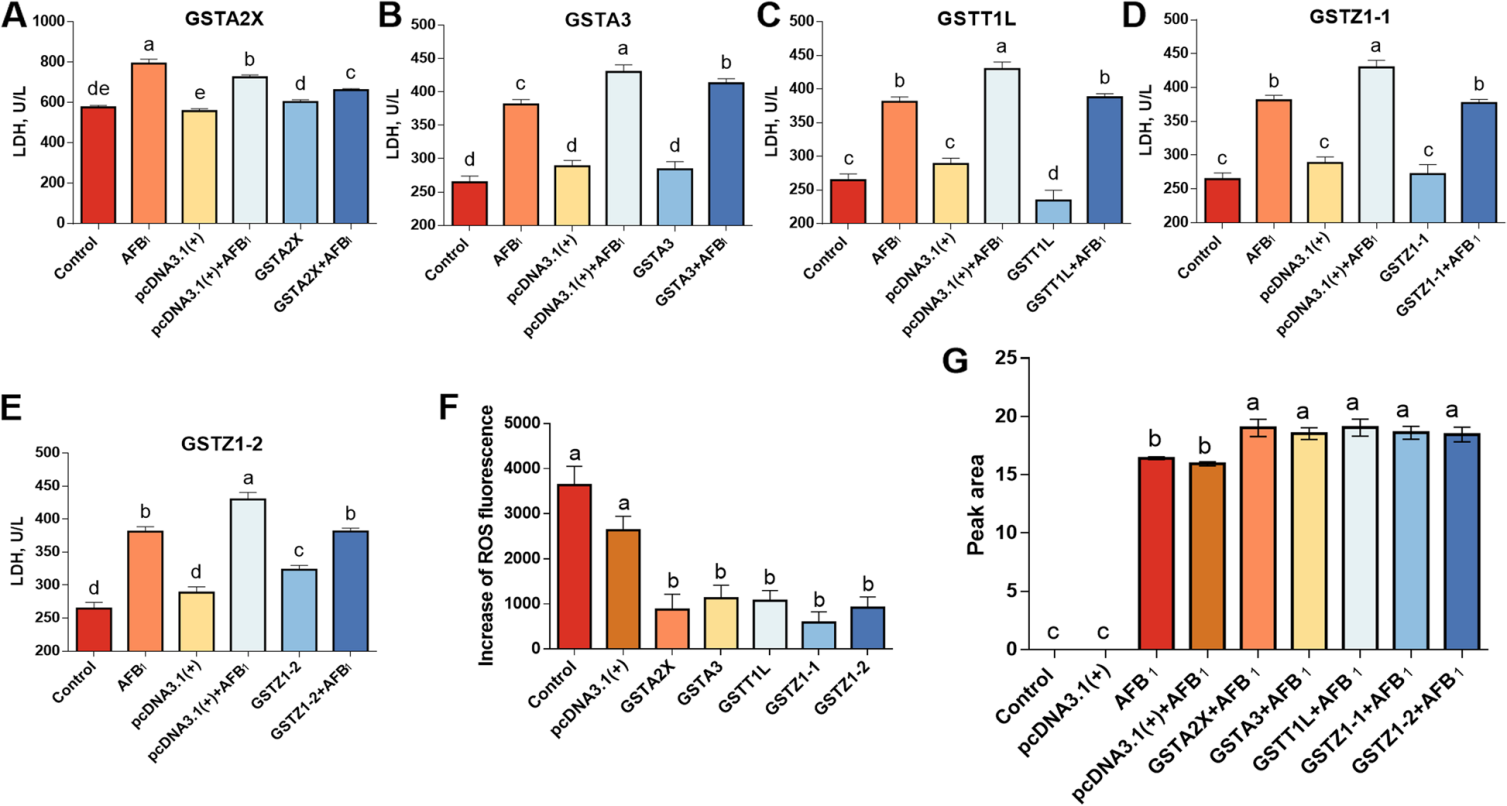
Effects of overexpressed GSTA2X, GSTA3, GSTT1L, GSTZ1-1, and GSTZ1-2 on the LDH release (**A**–**E**), ROS accumulation (**F**) and AFBO-GSH production (**G**) in LMH cell treated with AFB_1_. **A**–**E** Control, cells without any treatment; Control+AFB_1_, cells treated with 100 μg/L AFB_1_; pcDNA3.1(+), the cells transfected with pcDNA3.1(+) plasmid; pcDNA3.1(+) +AFB_1_, the cells transfected with pcDNA3.1(+) plasmid plus AFB_1_ treatment; GSTs, the cells transfected with different pcDNA3.1(+)-GST plasmid; GSTs+AFB_1_, the cells transfected with different pcDNA3.1(+)-GSTs plus AFB_1_ treatment. **F** Control, cells treated with 100 μg/L AFB_1_; pcDNA3.1(+), the cells transfected with pcDNA3.1(+) plasmid plus AFB_1_ treatment; GSTs, the cells transfected with different pcDNA3.1(+)-GSTs plus AFB_1_ treatment. **G** Control, cells without any treatment; pcDNA3.1(+), the cells transfected with pcDNA3.1(+) plasmid; AFB_1_, cells treated with 100 μg/L AFB_1_; pcDNA3.1(+) +AFB_1_, the cells transfected with pcDNA3.1(+) plasmid plus AFB_1_ treatment; GSTs+AFB_1_, the cells transfected with different pcDNA3.1(+)-GSTs plus AFB_1_ treatment. Values are expressed as mean ± SD (*n* = 6–8). ^a^^–^^e^Different letters between groups represent significant differences, *P* < 0.05

**Fig. 3:**
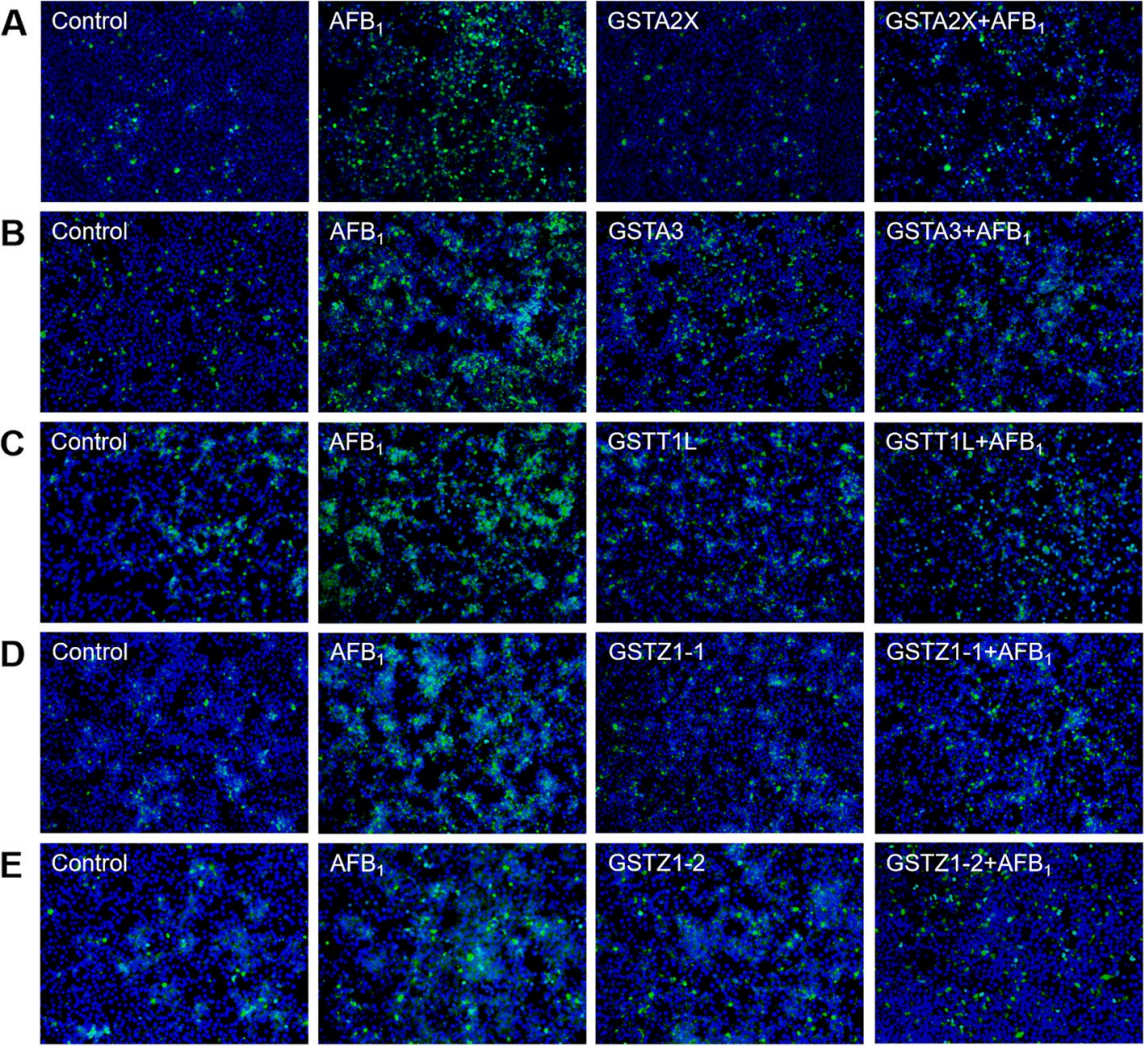
Effects of overexpressed GSTA2X (**A**), GSTA3 (**B**), GSTT1L (**C**), GSTZ1-1 (**D**) and GSTZ1-2 (**E**) in LMH cell treated with AFB_1_on DNA damage marker (*n* = 3). Control, cells without any treatment; AFB_1_, cells treated with 100 μg/L AFB_1_; GSTs, the cells transfected with different pcDNA3.1(+)-GSTs; GSTs+AFB_1_, the cells transfected with different pcDNA3.1(+)-GSTs plus AFB_1_ treatment

According to the results of cell viability, the present study further determined the LDH activity, intracellular ROS level, and DNA damage. Compared to the control and pcDNA3.1(+) group, AFB_1_ increased (*P* < 0.05) the LDH content by 37.7%–44.1% and 30.0%–49.0% in the supernatant of cell culture medium, respectively; while overexpression of GSTA2X, GSTA3, GSTZ1-1 and GSTZ1-2 reduced (*P* < 0.05) the elevated LDH release induced by AFB_1_ by 64.4%, 8.8%, 25.8% and 59.0% (Fig. 2A–E). Meanwhile, overexpressed GSTA2X, GSTA3, GATT1L, GSTZ1-1, and GSTZ1-2 decreased (*P* < 0.05) the intracellular ROS level by 57.2%–77.6% compared to the group transfected with blank pcDNA3.1(+) (Fig. 2F). At 24 h post-transfection, the cells were treated with AFB_1_ for 24 h and then AFBO-GSH was extracted and quantified, which showed a significant increase in the production of AFBO-GSH in the AFB_1_-treated group (Fig. 2G). Compared to the group transfected with blank vectors, the overexpression of GSTA2X, GSTA3, GATT1L, GSTZ1-1, and GSTZ1-2 increased (*P* < 0.05) the production of AFBO-GSH by 19.4%, 16.3%, 19.6%, 16.8%, and 15.8%, respectively (Fig. 2G). As shown in Fig. 3A–E, AFB_1_ led to DNA damage of LMH cells illustrated by the increase of γ-H2AX-positive cells, while overexpression of 5 GST isozymes alleviated the DNA damage.

### Sequences alignment and phylogenetic analysis of chicken GST proteins

**Fig. 4:**
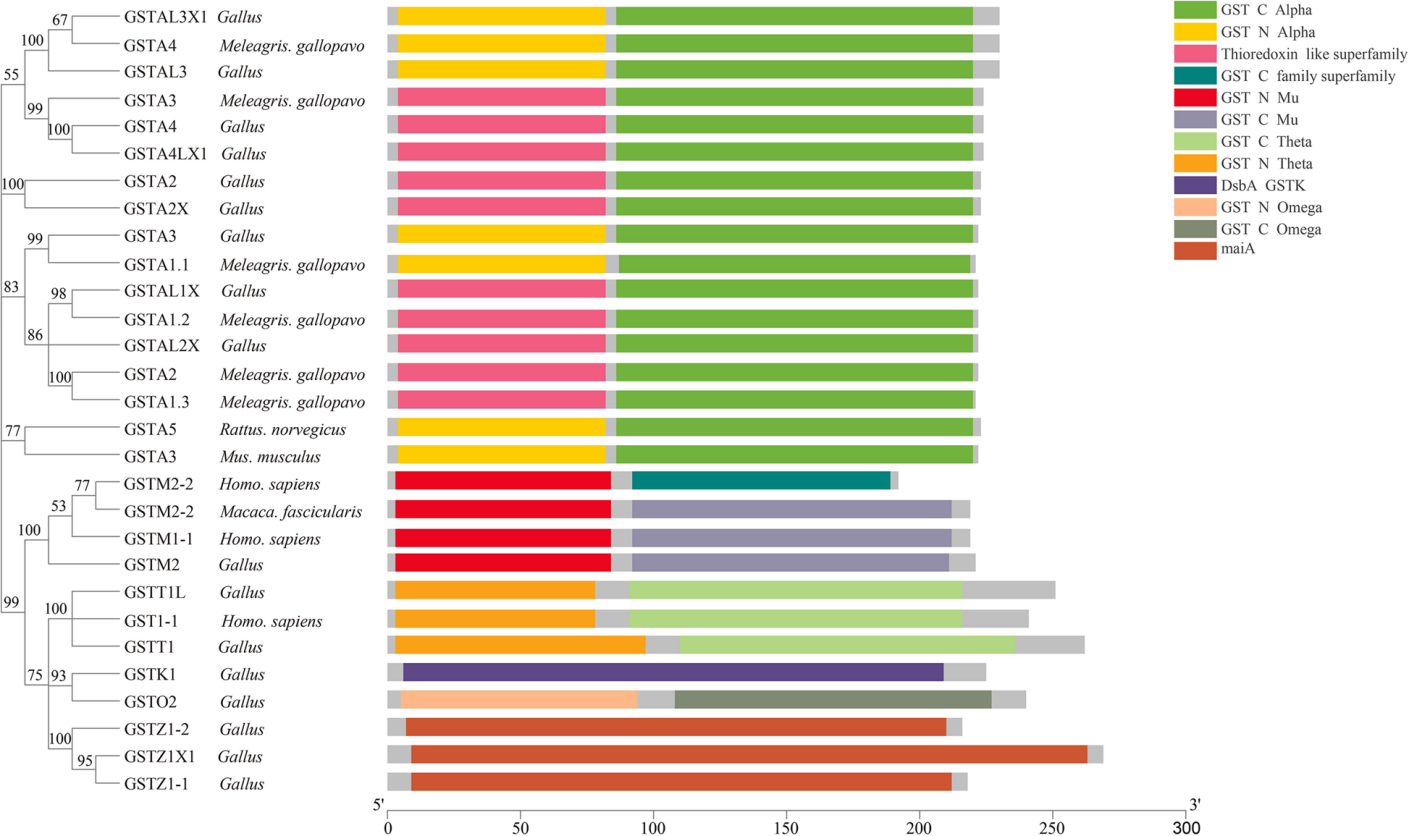
Phylogenetic analysis of amino acid sequences of chicken GSTs along with other five species *(Meleagris gallopavo*, *Homo sapiens*, *Mus musculus*, *Rattus norvegicus*, *Macaca fascicularis*) GSTs from NCBI. Unrooted trees were constructed by the maximum likelihood method. Bootstrap supporting values are indicated at each node. Different color rectangles mean different protein domains. GST, glutathione *S*-transferase

Seventeen GST proteins of *Gallus gallus* and the other twelve GST proteins (from *Meleagris gallopavo*, *Rattus norvegicus*, *Mus musculus*, *Macaca fascicularis,* and *Homo sapiens*) were aligned to analyze the homologies among them. As shown in Additional file 3: Fig. S1, these GST proteins shared some highly conserved amino acid sites even though they were from different species. Notably, GSTA2X and GSTA3 had more conversed amino acid sequences than GSTT1L, GSTZ1-1 and GSTZ1-2. Phylogenic tree and domain diagrams of the GST protein sequences were constructed to illustrate the phylogenetic relationships among them (Fig. 4). The phylogenetic tree classified the GST proteins into five subclades (subclade 1–4, GSTAs from different species; while subclade 5 included GSTMs, GSTTs, GSTK1, GSTO2, and GSTZs). As shown in Fig. 4, nine GSTAs possessed GST N Alpha or Thioredoxin like superfamily and GST C Alpha domain, GSTM2 possessed GST N Mu and GST C Mu domain, GSTT1L and GSTT1 possessed GST N Theta and GST C Theta domain, and GSTO2 possessed GST N Omega and GST C Omega domain, while the other four GSTs possessed special domain. In total, all GSTAs, GSTM and GSTTs possessed typical GST N-terminal and GST C-terminal. The coverage and identity of 17 GST amino acid sequences of *Gallus gallus* were compared with the *Mus muculus* GSTA3. As shown in Additional file 4: Table S3, the coverage of these five GSTs was 100.00%, 100.00%, < 30.00%, 82.11%, and 81.94% respectively, while their identity was 77.83%, 78.64%, < 30.00%, 43.08%, and 43.01%, respectively.

### Cloning, expression and purification of the reGSTA2X

**Fig. 5:**
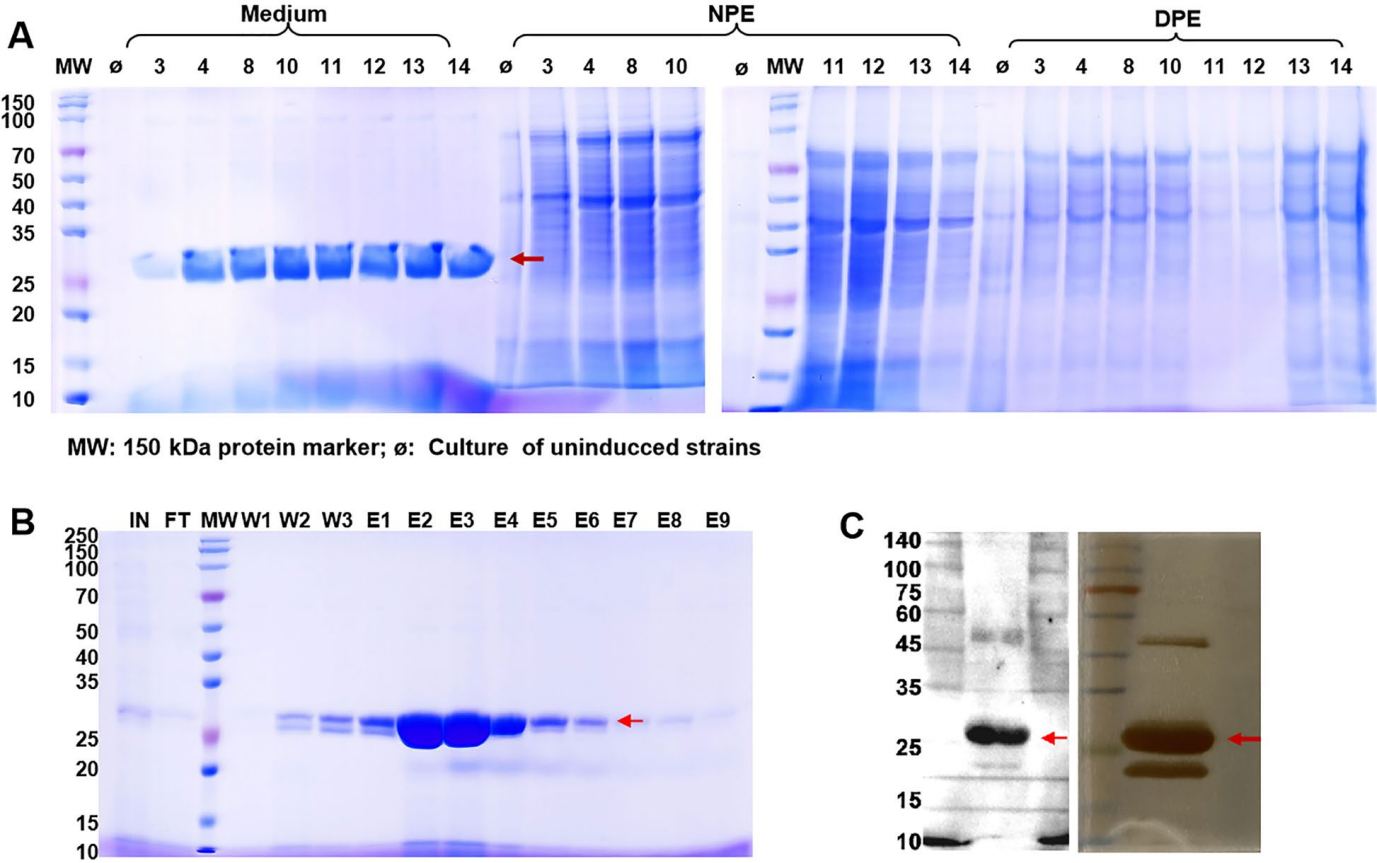
Expression and purification of the recombinant GSTA2X. **A** The SDS-PAGE determination of culture supernatant from positive single clones. **B** Purification of the recombinant GSTA2X. **C** Silver staining and Western Blot results of the purified reGSTA2X. The red arrow indicated the location of reGSTA2X

The pPICZαA-GSTA2X vector and its linearized vector (Additional file 5: Fig. S2A) and the selection of positive single clones (Additional file 5: Fig. S2B) were shown on the 1% agarose gel. After 72 h induction by 1% methanol, the target protein was detected in the culture medium supernatant when determined by 10% SDS-PAGE (Fig. 5A). The purified reGSTA2X also showed a clear band, visualized by staining with Coomassie Brilliant Blue and silver staining reagent, with a molecular size at approximately 25 kDa and it was further verified by Western-Blot (Fig. 5 B and C).

### Characterization of the reGSTA2X

**Fig. 6:**
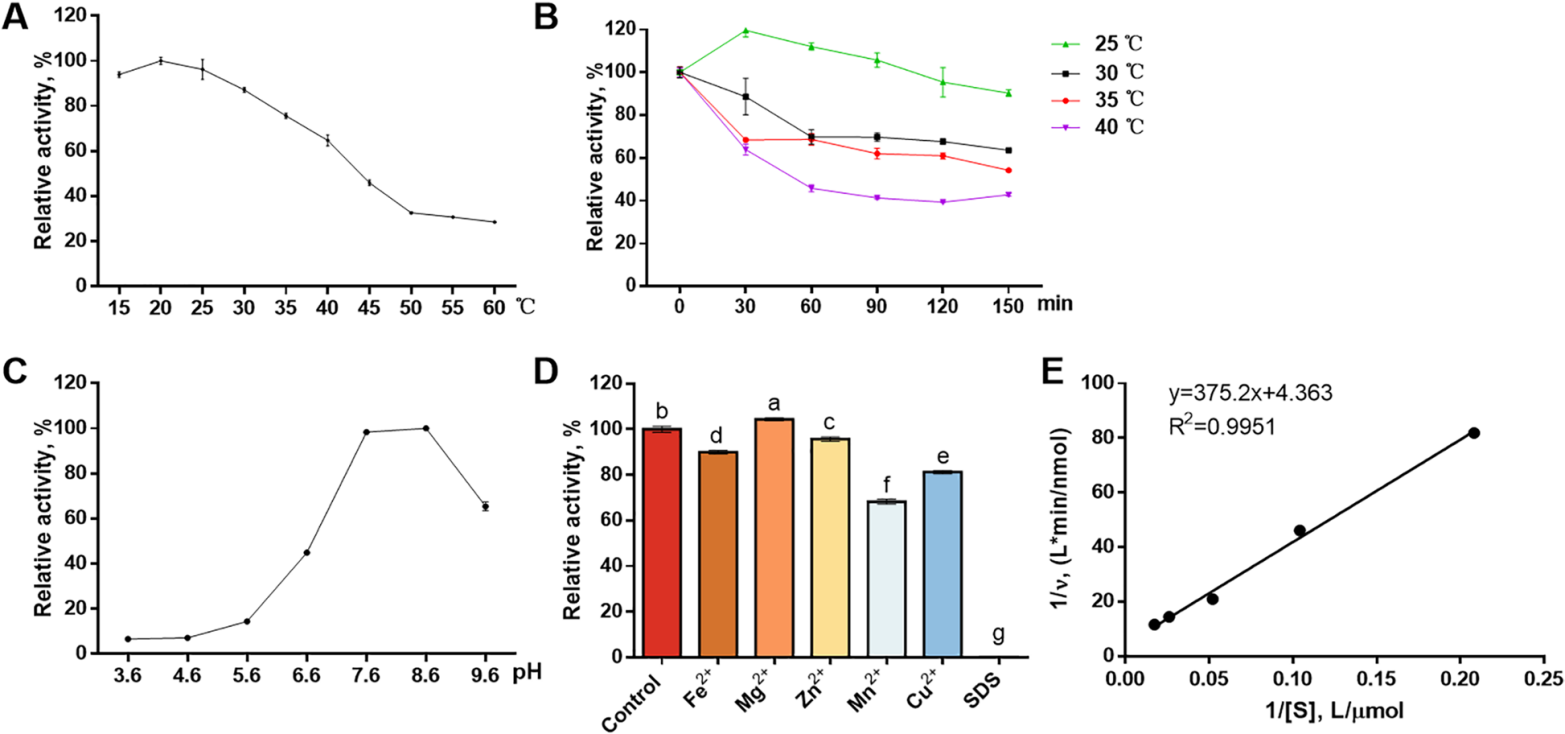
The enzymatic characteristics and parameters of reGSTA2X. **A** Effects of temperature on reGSTA2X activity. **B** The thermostability of reGSTA2X at different temperatures was determined by preincubating the enzyme at these temperatures in the absence of substrate for 30, 60, 90, 120, and 150 min before measuring its activity. **C** Effects of pH on reGSTA2X activity. **D** Effects of metal ion, and sodium dodecyl sulfonate (SDS) on reGSTA2X activity. **E** The enzyme kinetic curve of reGSTA2X. *K*_*m*_ = Michaelis constant, the substrate concentration at which the reaction velocity is 50% of the *V*_*max*_; *V* = reaction velocity; *V*_*max*_ = maximal reaction velocity; S = substrate concentration

The temperature-activity, pH-activity, and thermal stability of reGSTA2X were shown in Fig. 6. The reGSTA2X maintained more than 60% of its enzymatic activity at temperature between 15 and 40 °C, with 20 °C as the optimal temperature (Fig. 6A). Incubating the reGSTA2X at 25 °C for 60 min showed no impact on its enzymatic activity; furthermore, the activity of reGSTA2X kept more than 60% when it was incubated at 25 to 35 °C for 30 min, while incubating the enzymes at 40 °C for more than 30 min resulted in 50% activity loss (Fig. 6B). Moreover, the optimal pH of the reGSTA2X was 7.5 to 8.5 (Fig. 6C). The Fe^2+^, Zn^2+^, Mn^2+^, and Cu^2+^ at the concentration of 1 mmol/L inhibited the activity of reGSTA2X by 4.28%−31.73%, while 0.1% SDS completely inactivated the enzymes. In contrast, the activity of the enzymes increased by 4.36% in the presence of Mg^2+^ (Fig. 6D). The purified reGSTA2X showed a *V*_*max*_ for AFB_1_ as 0.23 nmol/min/mg protein and *K*_*m*_ as 86.05 μmol/L (Fig. 6E).

## Discussion

GSTs are the most important phase II enzymes in the detoxification of AFB_1_ in the liver. Poultry, especially the young, are more sensitive to AFB_1_ than other mammals due to the low catalyzation activity of GST enzymes [18]. Previous studies showed that GSTs enzymes catalyzing the conjugation of AFBO with GSH in other animals are mainly the GSTα, GSTμ and GSTθ sub-families [14, 24, 25]. In this study, five overexpressed GST isozymes (GSTA2X, GSTA3, GSTT1L, GSTZ1-1, and GSTZ1-2) of chicken alleviated the AFB_1_-induced death of LMH cell, which could be attributed to their ability in decreasing LDH activity and DNA damage as well as increasing AFBO-GSH production. The other twelve GST isozymes showed no mitigation effects on AFB_1_-induced toxicity, which might be due to the excessive GST activity depleting intracellular GSH so that its negative effects outweighed its weak detoxification ability [38].

LDH is a kind of significant enzyme involved in anaerobic glycolysis and gluconeogenesis, the increase of which reflects abnormal cell morphology, vitality, and migration ability [39, 40]. When the cell suffers from damage, its membranes usually lose integrity and the intracellular LDH subsequently gets released outside [41]. AFB_1_ could increase the LDH release both in vitro and in vivo according to Zhang et al. [5] and Altyar et al. [42]. Consistent with previous studies, we noticed that AFB_1_ increased the LDH content by 30.0%−49.0% in the supernatant of the cell culture medium in the present study. While the overexpression of GSTA2X, GSTA3, GSTZ1-1, and GSTZ1-2 downgraded the content of LDH by 8.8%–64.4%, suggesting their mitigation effects on abnormal cell morphology, damage or death. ROS are generally considered by-products of aerobic respiration, formed by the partial reduction of molecular oxygen [43, 44]. ROS accumulation will damage to DNA, protein and lipid, thus promoting genetic instability and tumorigenesis [45–47]. Xenogeneic AFB_1_ typically leads to oxidative stress via the elevation of ROS production [5, 48, 49]. Notably, five overexpressed GST isozymes reduced the typical accumulation of ROS, which suggested that these isozymes could glutathionylate ROS-generating enzymes with GSH, thus inhibiting superoxide and hydrogen peroxide in both mitochondria and the cytoplasm [50]. This mechanism explains the ability of the five GST isozymes in reducing ROS production.

AFBO-GSH is the most critical metabolite in AFB_1_ detoxification pathway in the liver of chicken [51]. Gamma-H2AX, generated from the rapid phosphorylation of H2AX histone protein at the serine-139 position in early response to a broad range of DNA lesions, is a highly effective biomarker for DNA strand breaks and DNA oxidation and plays a central role in sensing and repairing DNA damage [52–54]. AFB_1_ induced the expression of gamma-H2AX in LMH cells in this study, while five overexpressed GST isozymes decreased the positive gamma-H2AX cells; indicating mitigation effects on DNA lesion as Mo et al. reported [55]. In the present study, AFB1-induced DNA damage resulted from the elevation of ROS which can directly oxidize nucleoside bases, leading to G-T or G-A transversions as Srinivas et al. reported [56]. Additionally, the highly reactive and electrophilic AFBO attacks DNA at the N7 position on guanine, leading to the DNA lesion [19]. Thereby, overexpression of GSTA2X, GSTA3, GSTT1L, GSTZ1-1 and GSTZ1-2 could mitigate DNA damage because of their positive effects on eliminating ROS and AFBO.

Taken together, if the five isozymes were sorted according to their ability in mitigating the abnormal increase in LDH, ROS, DNA damage, as well as their facilitation effects on the production of AFBO-GSH, the GSTA2X had superior performance compared to GSTA3, GSTT1L, GSTZ1-1 and GSTZ1-2. In addition, the coverage and identity of GSTA2X compared to GSTA3 in mice were 100.00% and 77.83%, respectively; the latter exhibited the highest catalytic efficiency in the reaction of AFBO and GSH among other animals, which was 5–1,000 times higher than GSTs in rat and human liver [14, 57]. GSTA2X and GSTA3, as dimeric enzymes, were made of two distinct domains: a N-terminal thioredoxin-like domain and a C-terminal alpha-helical domain; the former domain was responsible for specific binding with GSH at G-site [22]. In summary, the crucial GSTA2X was selected and expressed in *P. pastoris* X-33.

The enzymatic parameters of reGSTA2X were determined using an incubation system with AFB_1_, GSH, microsomal and NADPH. Precisely, the optimal temperature and pH of reGSTA2X were 20–25 °C and 7.6–8.6, respectively. Previous studies have reported that the optimal temperature and pH of GSTS1, GSTS2, and GSTK1 in *Haliotis discus discus* were 30–40 °C and 8.0–9.0, while those of GSTK1 in *Hippocampus abdominalis* were 30 °C and 7.0–8.0 [58–60]. Although the optimal temperature showed difference, to a lesser extent, between reGSTA2X and other GSTs in aforementioned species, which might be due to the interspecies diversity [58–60]. Generally, the reaction temperature and pH were 37–39 °C and 7.4–7.6 in the exploring trial involved in the catalytic efficiency of GSTs [25, 61], while reGSTA2X maintained more than 70% of its catalytic activity at 15–37 °C. Moreover, the concentrations of Fe^2+^, Zn^2+^, Mn^2+^, Cu^2+^, and Mg^2+^ were 2.32–2.77, 0.34–4.78, 0.046–0.065, 0.047–0.190 and 8.76 mmol/L, respectively, in the chicken liver in accordance with previous studies [62–64]. Although 1 mmol/L Fe^2+^ and Zn^2+^ were normal according to previous reports, it still exhibited 4.3%−10.1% inhibition in the enzymatic activity. The negative effects of Mn^2+^ and Cu^2+^ on reGSTA2X might be due to their high dose far exceeding the normal concentration in liver. However, Mg^2+^ facilitated the catalytic activity of reGSTA2X in the reaction system, suggesting that Mg^2+^ might participate in catalysis. In the current study, the estimated *V*_*max*_ and *K*_*m*_ were 0.23 nmol/min/mg and 86.05 μmol/L, while Murcia and Diaz reported that GSTs extracted from liver of Rhode Island Red chickens displayed *V*_*max*_ as 1.40 pmol/min/mg and *K*_*m*_ as 65.66 μmol/L in the formation of AFBO-GSH [31]. The reGSTA2X showed higher *V*_*max*_ and *K*_*m*_ compared to GSTs from Rhode Island Red chickens, which might be attributed to the higher purity of reGSTA2X and variety differences of these two GSTs.

## Conclusion

Taken together, the present study identified five GST isozymes, GSTA2X, GSTA3, GSTT1L, GSTZ1-1, and GSTZ1-2, played key roles in detoxifying AFB_1_ in chicken. Specifically, these five GST isozymes could increase the cell viability, reduce the LDH release and ROS accumulation, and facilitate the production of AFBO-GSH, thus alleviating AFB_1_-induced DNA damage in the LMH cells. After comprehensive evaluation of the aforementioned indicators, GSTA2X displayed the best detoxification effects against AFB_1_. Thus, the enzymatic characteristics of GSTA2X were further analyzed and showed the *V*_*max*_ as 0.23 nmol/min/mg and *K*_*m*_ as 86.05 μmol/L with the AFB_1_ as substrate. Future work should be focused on exploring nutritional strategies that could regulate those pivotal GST isozymes to remediate the aflatoxicosis in chickens.

## Supplementary Information

Additional file 1: Table S1. Primers for the construction of 17 GST expression vectors.

Additional file 2: Table S2. Primers for qPCR of 17 GST isozyme genes.

Additional file 3: Fig. S1. Homologies analysis between 17 GST proteins of *Gallus gallus* and 12 GST proteins from *Meleagris gallopavo*, *Rattus norvegicus*, *Mus musculus*, *Macaca fascicularis* and *Homo sapiens*.

Additional file 4: Table S3. The coverage and identity of 17 GST isozymes compared to GSTA3 of *Mus musculus*.

Additional file 5: Fig. S2. The construction of pPICZαA-GSTA2X vector (A) and selection of positive single clones (B).

## Data Availability

Data will be made available on request.

## Abbreviations

AFB_1_: Aflatoxin B_1_
AFBO: AFB_1_-8,9-exo-epoxide
CYP450: Cytochrome P450
GST: Glutathione *S*-transferase
IARC: International Agency for Research on Cancer
LDH: Lactate dehydrogenase
LMH: Chicken Leghorn male hepatoma
NCBI: National Center for Biotechnology Information
ROS: Reactive oxygen species
SDS-PAGE: SDS-polyacrylamide gel electrophoresis

## References

[1] Jiang S, Xu L, Chen Y, Shu Z, Lv L, Zhao Y, et al. Longitudinal gut fungal alterations and potential fungal biomarkers for the progression of primary liver disease. Sci China Life Sci. 2024;67(6):1183–98. 10.1007/s11427-023-2458-1. 38413553 10.1007/s11427-023-2458-1

[2] Zhao L, Feng Y, Xu ZJ, Zhang NY, Zhang WP, Zuo G, et al. Selenium mitigated aflatoxin B_1_-induced cardiotoxicity with potential regulation of 4 selenoproteins and ferroptosis signaling in chicks. Food Chem Toxicol. 2021;154:112320. 10.1016/j.fct.2021.112320. 34116104 10.1016/j.fct.2021.112320

[3] Liu M, Zhao L, Gong G, Zhang L, Shi L, Dai J, et al. Invited review: remediation strategies for mycotoxin control in feed. J Anim Sci Biotechnol. 2022;13:19. 10.1186/s40104-021-00661-4. 35090579 10.1186/s40104-021-00661-4 PMC8796454

[4] Zhao L, Feng Y, Deng J, Zhang NY, Zhang WP, Liu XL, et al. Selenium deficiency aggravates aflatoxin B_1_-induced immunotoxicity in chick spleen by regulating 6 selenoprotein genes and redox/inflammation/apoptotic signaling. J Nutr. 2019;149(6):894–901. 10.1093/jn/nxz019. 31070734 10.1093/jn/nxz019

[5] Zhang Y, Cao KX, Niu QJ, Deng J, Zhao L, Khalil MM, et al. Alpha-class glutathione S-transferases involved in the detoxification of aflatoxin B_1_ in ducklings. Food Chem Toxicol. 2023;174:113682. 10.1016/j.fct.2023.113682. 36813151 10.1016/j.fct.2023.113682

[6] Zhao L, Deng J, Ma LB, Zhang WP, Khalil MM, Karrow NA, et al. Dietary Se deficiency dysregulates metabolic and cell death signaling in aggravating the AFB_1_ hepatotoxicity of chicks. Food Chem Toxicol. 2021;149:111938. 10.1016/j.fct.2020.111938. 33348051 10.1016/j.fct.2020.111938

[7] Kos J, Anić M, Radić B, Zadravec M, Janić Hajnal E, Pleadin J. Climate change-A global threat resulting in increasing mycotoxin occurrence. Foods. 2023;12(14):2704. 10.3390/foods12142704. 37509796 10.3390/foods12142704 PMC10379110

[8] Carey CN, Paquette M, Sahye-Pudaruth S, Dadvar A, Dinh D, Khodabandehlou K, et al. The environmental sustainability of plant-based dietary patterns: a scoping review. J Nutr. 2023;153(3):857–69. 10.1016/j.tjnut.2023.02.001. 36809853 10.1016/j.tjnut.2023.02.001

[9] Cao KX, Deng ZC, Li SJ, Yi D, He X, Yang XJ, et al. Poultry nutrition: achievement, challenge, and strategy. J Nutr. 2024;S0022–3166(24):01107–16. 10.1016/j.tjnut.2024.10.030. 10.1016/j.tjnut.2024.10.030 39424066

[10] Liu X, Kumar Mishra S, Wang T, Xu Z, Zhao X, Wang Y, et al. AFB_1_ induced transcriptional regulation related to apoptosis and lipid metabolism in liver of chicken. Toxins (Basel). 2020;12(5):290. 10.3390/toxins12050290. 32375309 10.3390/toxins12050290 PMC7290437

[11] Zhao L, Zhang L, Xu Z, Liu X, Chen L, Dai J, et al. Occurrence of aflatoxin B_1_, deoxynivalenol and zearalenone in feeds in China during 2018–2020. J Anim Sci Biotechnol. 2021;12:74. 10.1186/s40104-021-00603-0. 34243805 10.1186/s40104-021-00603-0 PMC8272344

[12] Deng J, Huang JC, Xu ZJ, Liu Y, Karrow NA, Liu M, et al. Remediation strategies for mycotoxins in animal feed. Toxins (Basel). 2023;15(9):513. 10.3390/toxins15090513. 37755939 10.3390/toxins15090513 PMC10535302

[13] Liu S, Kang W, Mao X, Ge L, Du H, Li J, et al. Melatonin mitigates aflatoxin B_1_-induced liver injury via modulation of gut microbiota/intestinal FXR/liver TLR4 signaling axis in mice. J Pineal Res. 2022;73(2):e12812. 10.1111/jpi.12812. 35652241 10.1111/jpi.12812

[14] Deng J, Yang JC, Feng Y, Xu ZJ, Kuča K, Liu M, et al. AP-1 and SP1 trans-activate the expression of hepatic CYP1A1 and CYP2A6 in the bioactivation of AFB_1_ in chicken. Sci China Life Sci. 2024;67(7):1468–78. 10.1007/s11427-023-2512-6. 38703348 10.1007/s11427-023-2512-6

[15] Sun LH, Zhang NY, Zhu MK, Zhao L, Zhou JC, Qi DS. Prevention of aflatoxin B_1_ hepatoxicity by dietary selenium is associated with inhibition of cytochrome P450 isozymes and up-regulation of 6 selenoprotein genes in chick liver. J Nutr. 2015;146(4):655–61. 10.3945/jn.115.224626. 26962192 10.3945/jn.115.224626

[16] Zhang NY, Qi M, Zhao L, Zhu MK, Guo J, Liu J, et al. Curcumin prevents aflatoxin B₁ hepatoxicity by inhibition of cytochrome P450 isozymes in chick liver. Toxins (Basel). 2016;8(11):327. 10.3390/toxins8110327. 27834912 10.3390/toxins8110327 PMC5127124

[17] Liu Y, Zheng Z. Mitigation of understanding the global cancer statistics 2022: growing cancer burden. Sci China Life Sci. 2024;67(10):2274–6. 10.1007/s11427-024-2657-y. 39136859 10.1007/s11427-024-2657-y

[18] Deng J, Zhao L, Zhang N-Y, Karrow NA, Krumm CS, Qi D-S, et al. Aflatoxin B_1_ metabolism: regulation by phase I and II metabolizing enzymes and chemoprotective agents. Mutat Res Rev Mutat Res. 2018;778:79–89. 10.1016/j.mrrev.2018.10.002. 30454686 10.1016/j.mrrev.2018.10.002

[19] Rushing BR, Selim MI. Aflatoxin B_1_: a review on metabolism, toxicity, occurrence in food, occupational exposure, and detoxification methods. Food Chem Toxicol. 2019;124:81–100. 10.1016/j.fct.2018.11.047. 30468841 10.1016/j.fct.2018.11.047

[20] Ma M, Wang Q, Liu Y, Li G, Liu L, Wang G, et al. *Bacillus* CotA laccase improved the intestinal health, amino acid metabolism and hepatic metabolic capacity of Pekin ducks fed naturally contaminated AFB_1_ diet. J Anim Sci Biotechnol. 2024;15:138. 10.1186/s40104-024-01091-8. 39385285 10.1186/s40104-024-01091-8 PMC11465776

[21] Marchese S, Polo A, Ariano A, Velotto S, Costantini S, Severino L. Aflatoxin B_1_ and M_1_: biological properties and their involvement in cancer development. Toxins (Basel). 2018;10(6):214. 10.3390/toxins10060214. 29794965 10.3390/toxins10060214 PMC6024316

[22] Allocati N, Masulli M, Di Ilio C, Federici L. Glutathione transferases: substrates, inihibitors and pro-drugs in cancer and neurodegenerative diseases. Oncogenesis. 2018;7:8. 10.1038/s41389-017-0025-3. 29362397 10.1038/s41389-017-0025-3 PMC5833873

[23] Oakley A. Glutathione transferases: a structural perspective. Drug Metab Rev. 2011;43(2):138–51. 10.3109/03602532.2011.558093. 21428697 10.3109/03602532.2011.558093

[24] Crawford DR, Ilic Z, Guest I, Milne GL, Hayes JD, Sell S. Characterization of liver injury, oval cell proliferation and cholangiocarcinogenesis in glutathione S-transferase A3 knockout mice. Carcinogenesis. 2017;38(7):717–27. 10.1093/carcin/bgx048. 28535182 10.1093/carcin/bgx048 PMC5862260

[25] Kim JE, Bunderson BR, Croasdell A, Reed KM, Coulombe RA Jr. Alpha-class glutathione S-transferases in wild turkeys (*Meleagris gallopavo*): characterization and role in resistance to the carcinogenic mycotoxin aflatoxin B_1_. PLoS ONE. 2013;8(4):e60662. 10.1371/journal.pone.0060662. 23613737 10.1371/journal.pone.0060662 PMC3628786

[26] Yang JC, Liu M, Huang RH, Zhao L, Niu QJ, Xu ZJ, et al. Loss of SELENOW aggravates muscle loss with regulation of protein synthesis and the ubiquitin-proteasome system. Sci Adv. 2024;10(38):eadj4122. 10.1126/sciadv.adj4122. 39303039 10.1126/sciadv.adj4122 PMC11414739

[27] Zhao L, Liu M, Sun H, Yang JC, Huang YX, Huang JQ, et al. Selenium deficiency-induced multiple tissue damage with dysregulation of immune and redox homeostasis in broiler chicks under heat stress. Sci China Life Sci. 2023;66(9):2056–69. 10.1007/s11427-022-2226-1. 36795182 10.1007/s11427-022-2226-1

[28] Wang D, Kuang Y, Lv Q, Xie W, Xu X, Zhu H, et al. Selenium-enriched *Cardamine violifolia* protects against sepsis-induced intestinal injury by regulating mitochondrial fusion in weaned pigs. Sci China Life Sci. 2023;66(9):2099–111. 10.1007/s11427-022-2274-7. 36814047 10.1007/s11427-022-2274-7

[29] Lin J, Li X, Lu K, Song K, Wang L, Dai W, et al. Low phosphorus causes hepatic energy metabolism disorder through dynamin-related protein 1-mediated mitochondrial fission in fish. J Nutr. 2024;S0022–3166(24):01121. 10.1093/jn/nxx062. 10.1016/j.tjnut.2024.10.044 39491675

[30] Li H, Gao W, Wang H, Zhang H, Huang L, Yuan T, et al. Evidence from an avian embryo model that zinc-inducible MT4 expression protects mitochondrial function against oxidative stress. J Nutr. 2024;154(3):896–907. 10.1016/j.tjnut.2024.01.011. 38301957 10.1016/j.tjnut.2024.01.011

[31] Murcia HW, Diaz GJ. Protective effect of glutathione S-transferase enzyme activity against aflatoxin B_1_ in poultry species: relationship between glutathione S-transferase enzyme kinetic parameters, and resistance to aflatoxin B_1_. Poult Sci. 2021;100(8):101235. 10.1016/j.psj.2021.101235. 34214746 10.1016/j.psj.2021.101235 PMC8258694

[32] Wang S, Cao H, Zhao CC, Wang Q, Wang D, Liu J, et al. Engineering biomimetic nanosystem targeting multiple tumor radioresistance hallmarks for enhanced radiotherapy. Sci China Life Sci. 2024;67(7):1398–412. 10.1007/s11427-023-2528-5. 38602587 10.1007/s11427-023-2528-5

[33] Tamura K, Stecher G, Kumar S. MEGA11: molecular evolutionary genetics analysis version 11. Mol Biol Evol. 2021;38(7):3022–7. 10.1093/molbev/msab120. 33892491 10.1093/molbev/msab120 PMC8233496

[34] Lu S, Wang J, Chitsaz F, Derbyshire MK, Geer RC, Gonzales NR, et al. CDD/SPARCLE: the conserved domain database in 2020. Nucleic Acids Res. 2020;48(D1):D265–8. 10.1093/nar/gkz991. 31777944 10.1093/nar/gkz991 PMC6943070

[35] Chen C, Chen H, Zhang Y, Thomas HR, Frank MH, He Y, et al. TBtools: an integrative toolkit developed for interactive analyses of big biological bata. Mol Plant. 2020;13(8):1194–202. 10.1016/j.molp.2020.06.009. 32585190 10.1016/j.molp.2020.06.009

[36] Xi C, Liu N, Liang F, Zhao X, Long J, Yuan F, et al. Molecular assembly of recombinant chicken type II collagen in the yeast *Pichia pastoris*. Sci China Life Sci. 2018;61(7):815–25. 10.1007/s11427-017-9219-4. 29388039 10.1007/s11427-017-9219-4

[37] Gu Y, Huang Y, Qiu Z, Xu Z, Li D, Chen L, et al. Vitamin B_2_ functionalized iron oxide nanozymes for mouth ulcer healing. Sci China Life Sci. 2020;63(1):68–79. 10.1007/s11427-019-9590-6. 31463739 10.1007/s11427-019-9590-6

[38] Potęga A, Kosno M, Mazerska Z. Novel insights into conjugation of antitumor-active unsymmetrical bisacridine C-2028 with glutathione: characteristics of non-enzymatic and glutathione S-transferase-mediated reactions. J Pharm Anal. 2021;11(6):791–8. 10.1016/j.jpha.2021.03.014. 35028185 10.1016/j.jpha.2021.03.014 PMC8740389

[39] Cuddihy J, Wu G, Ho L, Kudo H, Dannhorn A, Mandalia S, et al. Lactate dehydrogenase activity staining demonstrates time-dependent immune cell infiltration in human ex-vivo burn-injured skin. Sci Rep. 2021;11:21249. 10.1038/s41598-021-00644-5. 34711882 10.1038/s41598-021-00644-5 PMC8553775

[40] Guo J, Yan E, He L, Wang Y, Xiang Y, Zhang P, et al. Dietary supplementation with lauric acid improves aerobic endurance in sedentary mice via enhancing fat mobilization and glyconeogenesis. J Nutr. 2023;153(11):3207–19. 10.1016/j.tjnut.2023.09.006. 37696395 10.1016/j.tjnut.2023.09.006

[41] Klobucher KN, Badger R, Foxall T, Erickson PS. Short communication: effect of sodium butyrate, monensin, and butyric acid on the viability of Eimeria bovis sporozoites and their degree of damage to a bovine epithelial cell line. J Anim Sci. 2022;100(12):skac360. 10.1093/jas/skac360. 36315476 10.1093/jas/skac360 PMC9733496

[42] Altyar AE, Kensara OA, Sayed AA, Aleya L, Almutairi MH, Zaazouee MS, et al. Acute aflatoxin B_1_-induced hepatic and cardiac oxidative damage in rats: Ameliorative effects of morin. Heliyon. 2023;9(11):e21837. 10.1016/j.heliyon.2023.e21837. 38027731 10.1016/j.heliyon.2023.e21837 PMC10663918

[43] Moloney JN, Cotter TG. ROS signalling in the biology of cancer. Semin Cell Dev Biol. 2018;80:50–64. 10.1016/j.semcdb.2017.05.023. 28587975 10.1016/j.semcdb.2017.05.023

[44] Li J, Fu C, Feng B, Liu Q, Gu J, Khan MN, et al. Polyacrylic acid-coated selenium-doped carbon dots inhibit ferroptosis to alleviate chemotherapy-associated acute kidney injury. Adv Sci (Weinh). 2024;11(28):e2400527. 10.1002/advs.202400527. 38689508 10.1002/advs.202400527 PMC11267338

[45] Nakamura H, Takada K. Reactive oxygen species in cancer: current findings and future directions. Cancer Sci. 2021;112(10):3945–52. 10.1111/cas.15068. 34286881 10.1111/cas.15068 PMC8486193

[46] Yan YQ, Liu M, Xu ZJ, Xu ZJ, Huang YX, Li XM, et al. Optimum doses and forms of selenium maintaining reproductive health via regulating homeostasis of gut microbiota and testicular redox, inflammation, cell proliferation, and apoptosis in roosters. J Nutr. 2024;154(2):369–80. 10.1016/j.tjnut.2023.12.021. 38122845 10.1016/j.tjnut.2023.12.021

[47] Deng ZC, Wang J, Wang J, Yan YQ, Huang YX, Chen CQ, et al. Tannic acid extracted from gallnut improves intestinal health with regulation of redox homeostasis and gut microbiota of weaned piglets. Anim Res One Health. 2024;2(1):16–27. 10.1002/aro2.51.

[48] Li Q, Zhang M, Sun J, Li Y, Zu S, Xiang Y, et al. Porcine β-defensin-2 alleviates aflatoxin B_1_ induced intestinal mucosal damage via ROS-Erk1/2 signaling pathway. Sci Total Environ. 2023;905:167201. 10.1016/j.scitotenv.2023.167201. 37734607 10.1016/j.scitotenv.2023.167201

[49] Zhang B, Li M, Zhou G, Gu X, Xie L, Zhao M, et al. ZnO-NPs alleviate aflatoxin B_1_-induced hepatoxicity in ducklings by promoting hepatic metallothionein expression. Ecotoxicol Environ Saf. 2023;256:114826. 10.1016/j.ecoenv.2023.114826. 36989561 10.1016/j.ecoenv.2023.114826

[50] Letourneau M, Wang K, Mailloux RJ. Protein S-glutathionylation decreases superoxide/hydrogen peroxide production xanthine oxidoreductase. Free Radic Biol Med. 2021;175:184–92. 10.1016/j.freeradbiomed.2021.08.243. 34481042 10.1016/j.freeradbiomed.2021.08.243

[51] Wang X, Yang F, Na L, Jia M, Ishfaq M, Zhang Y, et al. Ferulic acid alleviates AFB_1_-induced duodenal barrier damage in rats via up-regulating tight junction proteins, down-regulating ROCK, competing CYP450 enzyme and activating GST. Ecotoxicol Environ Saf. 2022;241:113805. 10.1016/j.ecoenv.2022.113805. 35772360 10.1016/j.ecoenv.2022.113805

[52] Kinner A, Wu W, Staudt C, Iliakis G. Gamma-H2AX in recognition and signaling of DNA double-strand breaks in the context of chromatin. Nucleic Acids Res. 2008;36(17):5678–94. 10.1093/nar/gkn550. 18772227 10.1093/nar/gkn550 PMC2553572

[53] Kopp B, Khoury L, Audebert M. Validation of the γH2AX biomarker for genotoxicity assessment: a review. Arch Toxicol. 2019;93(8):2103–14. 10.1007/s00204-019-02511-9. 31289893 10.1007/s00204-019-02511-9

[54] Sedelnikova OA, Redon CE, Dickey JS, Nakamura AJ, Georgakilas AG, Bonner WM. Role of oxidatively induced DNA lesions in human pathogenesis. Mutat Res. 2010;704(1–3):152–9. 10.1016/j.mrrev.2009.12.005. 20060490 10.1016/j.mrrev.2009.12.005 PMC3074954

[55] Mo YX, Ruan ML, Wang J, Liu Y, Wu YY, Wang GL, et al. Mitigating the adverse effects of Aflatoxin B_1_ in LMH, IPEC-J2 and 3D4/21 cells by a novel integrated agent. Food Chem Toxicol. 2023;178:113907. 10.1016/j.fct.2023.113907. 37343715 10.1016/j.fct.2023.113907

[56] Srinivas US, Tan BWQ, Vellayappan BA, Jeyasekharan AD. ROS and the DNA damage response in cancer. Redox Biol. 2019;25:101084. 10.1016/j.redox.2018.101084. 30612957 10.1016/j.redox.2018.101084 PMC6859528

[57] Van Ness KP, McHugh TE, Bammler TK, Eaton DL. Identification of amino acid residues essential for high aflatoxin B_1_–8,9-epoxide conjugation activity in alpha class glutathione S-transferases through site-directed mutagenesis. Toxicol Appl Pharmacol. 1998;152(1):166–74. 10.1006/taap.1998.8493. 9772212 10.1006/taap.1998.8493

[58] Samaraweera AV, Sandamalika WMG, Liyanage DS, Lee S, Priyathilaka TT, Lee J. Molecular characterization and functional analysis of glutathione S-transferase kappa 1 (GSTκ1) from the big belly seahorse (*Hippocampus abdominalis*): elucidation of its involvement in innate immune responses. Fish Shellfish Immunol. 2019;92:356–66. 10.1016/j.fsi.2019.06.010. 31200074 10.1016/j.fsi.2019.06.010

[59] Sandamalika WMG, Priyathilaka TT, Liyanage DS, Lee S, Lim HK, Lee J. Molecular characterization of kappa class glutathione S-transferase from the disk abalone (*Haliotis discus discus*) and changes in expression following immune and stress challenges. Fish Shellfish Immunol. 2018;77:252–63. 10.1016/j.fsi.2018.03.058. 29621633 10.1016/j.fsi.2018.03.058

[60] Wan Q, Whang I, Lee J. Molecular cloning and characterization of three sigma glutathione S-transferases from disk abalone (*Haliotis discus discus*). Comp Biochem Physiol B Biochem Mol Biol. 2008;151(3):257–67. 10.1016/j.cbpb.2008.07.012. 18703158 10.1016/j.cbpb.2008.07.012

[61] Lozano MC, Diaz GJ. Microsomal and cytosolic biotransformation of aflatoxin B_1_ in four poultry species. Br Poult Sci. 2006;47(6):734–41. 10.1080/00071660601084390. 17190682 10.1080/00071660601084390

[62] He Y, Sun B, Li S, Sun X, Guo Y, Zhao H, et al. Simultaneous analysis 26 mineral element contents from highly consumed cultured chicken overexposed to arsenic trioxide by inductively coupled plasma mass spectrometry. Environ Sci Pollut Res Int. 2016;23(21):21741–21750. 10.1007/s11356-016-7318-5. 10.1007/s11356-016-7318-5 27522209

[63] Hu Y, Zhang W, Chen G, Cheng H, Tao S. Public health risk of trace metals in fresh chicken meat products on the food markets of a major production region in southern China. Environ Pollut. 2018;234:667–676. 10.1016/j.envpol.2017.12.006. 10.1016/j.envpol.2017.12.006 29227952

[64] Uluozlu OD, Tuzen M, Mendil D, Soylak M. Assessment of trace element contents of chicken products from Turkey. J Hazard Mater. 2009;163(2–3):982–7. 10.1016/j.jhazmat.2008.07.050. 18752893 10.1016/j.jhazmat.2008.07.050

