# Supplementary material for: Five glutathione S-transferase isozymes played crucial role in the detoxification of aflatoxin B1 in chicken liver
Source: J Anim Sci Biotechnol. 2025 Apr 8;16:54. doi: 10.1186/s40104-025-01189-7 (PMC11977921; doi:10.1186/s40104-025-01189-7)
Supplement: Supplementary file 1 — Additional file 1: Table S1. Primers for the construction of 17 GST expression vectors. [file 40104_2025_1189_MOESM1_ESM.docx]

**Additional file 1: Table S1.** Primers for the construction of 17 GST expression vectors

| **Primers** | **Sequence (5´→3´)** | |
| --- | --- | --- |
| *GSTAL1X*-F | GCTGGCTAGCGTTTAAACTTAAGCTTACGTGGAAACATGTCTGG | |
| *GSTAL1X*-R | GTGGATCCGAGCTCGGTACCAAGCTTTGCTCAACTGAAAATGGC | |
| *GSTA2*-F | GCTGGCTAGCGTTTAAACTTAAGCTTGCTCTGAAATCATGGCTG | |
| *GSTA2*-R | GTGGATCCGAGCTCGGTACCAAGCTTAGCCACATGATTAGAAACTGA | |
| *GSTA2X*-F | GCTGGCTAGCGTTTAAACTTAAGCTTGCTCTGAAATCATGGCTGGG | |
| *GSTA2X*-R | GCTGGCTAGCGTTTAAACTTAAGCTTATCTGTAGCAGCCACATGATT | |
| *GSTAL2X*-F | GTGGATCCGAGCTCGGTACCAAGCTTTGCTCAACTGAAAATGGC | |
| *GSTAL2X*-R | GCTGGCTAGCGTTTAAACTTAAGCTTACGTGGAAGCATGTCTGG | |
| *GSTA3*-F | GCTGGCTAGCGTTTAAACTTAAGCTTACGTGGGAACATGTCTGG | |
| *GSTA3*-R | GTGGATCCGAGCTCGGTACCAAGCTTTGTTCAGTGGAAAATTGC | |
| *GSTAL3*-F | GCTGGCTAGCGTTTAAACTTAAGCTTATGGCTGCAAAACCTGTA | |
| *GSTAL3*-R | GTGGATCCGAGCTCGGTACCAAGCTTACAAGCACGCTAATGTGG | |
| *GSTAL3X1*-F | GCTGGCTAGCGTTTAAACTTAAGCTTCCATGGCTGCAAAACCTGTA | |
| *GSTAL3X1*-R | GTGGATCCGAGCTCGGTACCAAGCTTGTGGATCCGAGCTCGGTACC | |
| *GSTA4*-F | GCTGGCTAGCGTTTAAACTTAAGCTTCTCAGAAGCATGTCGGGGAA | |
| *GSTA4*-R | GTGGATCCGAGCTCGGTACCAAGCTTTGAAGTTAACGCTGCACTCA | |
| *GSTA4LX1*-F | GCTGGCTAGCGTTTAAACTTAAGCTTAGAAGCATGTCGGGGAAG | |
| *GSTA4LX1*-R | GTGGATCCGAGCTCGGTACCAAGCTTACGCTGCACTCAGTTTAG | |
| *GSTM2*-F | GCTGGCTAGCGTTTAAACTTAAGCTTCTCGCCATGGTGGTCACG | |
| *GSTM2*-R | GTGGATCCGAGCTCGGTACCAAGCTTCCCCTCACTCTTTCTTGTTGTT | |
| *GSTK1*-F | | GCTGGCTAGCGTTTAAACTTAAGCTTTAACATGGGTCGGGTACT |
| *GSTK1*-R | | GTGGATCCGAGCTCGGTACCAAGCTTCCAGTACTTTTCACATTTT |
| *GSTO2*-F | | GCTGGCTAGCGTTTAAACTTAAGCTTCAGCACATTACAGCGGTAT |
| *GSTO2*-R | | GTGGATCCGAGCTCGGTACCAAGCTTTTTGGAGTATGAGTCAAAACA |
| *GSTT1*-F | | GCTGGCTAGCGTTTAAACTTAAGCTTATTCCGACGGAGCGTCCC |
| *GSTT1*-R | | GTGGATCCGAGCTCGGTACCAAGCTTTTCATTTCAACATCTTCAT |
| *GATT1L*-F | | GCTGGCTAGCGTTTAAACTTAAGCTTCAGCATGGGGCTGGAGCT |
| *GATT1L*-R | | GTGGATCCGAGCTCGGTACCAAGCTTGCTGGTTGCAGTCAAAGAT |

Note: The underlined sequence was the homology arms of pc DNA3.1(+)
