# Supplementary material for: Five glutathione S-transferase isozymes played crucial role in the detoxification of aflatoxin B1 in chicken liver
Source: J Anim Sci Biotechnol. 2025 Apr 8;16:54. doi: 10.1186/s40104-025-01189-7 (PMC11977921; doi:10.1186/s40104-025-01189-7)
Supplement: Supplementary file 2 — Additional file 2: Table S2. Primers for qPCR of 17 GST isozyme genes. [file 40104_2025_1189_MOESM2_ESM.docx]

**Additional file 2: Table S2.** Primers for qPCR of 17 GST isozyme genes

| **Genes** | **Forward primers (5’-3’)** | **Reverse primers (5’-3’)** |
| --- | --- | --- |
| *GSTAL1X* | tgtatgtggaaggactggca | acaagaaagtcatgcccgtg |
| *GSTA2* | GATGGAGTCAATTCGGTGGC | GCACCATCTTCATCCCATCG |
| *GSTA2X* | cgatgggatgaagatggtgc | ctttcacatcggctgcttga |
| *GSTAL2X* | acaaggccgcaaacagatac | gaggtggtttctttgggctg |
| *GSTA3* | CACTATGCCAACACACGAGG | GGGCTCTCTCCTTCAGATCC |
| *GSTAL3* | ggccaggactttcttgttgg | gaaccctcctcacagtctcc |
| *GSTAL3X1* | ggccaggactttcttgttgg | gaaccctcctcacagtctcc |
| *GSTA4* | agagagccctgatcgacatg | gtcttggccatgctgtttca |
| *GSTA4LX1* | tatgtcaatgggagagggcg | accaacttcatcccgtcgat |
| *GSTM2* | GGTCACGTTGGGTTATTGGG | ATGAGATAGGGCAGGTTGGG |
| *GSTK1* | tcgaggttctctgccgatac | tgataaaacgcatggctccc |
| *GSTO2* | tatgcgtttctgcccctttg | tctcatacgggtctgaaggc |
| *GSTT1* | aggtgctgattcccctcttc | ctgccaaggaaatctcgctc |
| *GSTT1L* | cccgatcactggtatccctc | cccgatcactggtatccctc |
| *GSTZ1X1* | cagaagaataccgtggcagc | tcatctcccacacagtagcg |
| *GSTZ1-1* | ctatgaccaggtgccagtga | ctatgaccaggtgccagtga |
| *GSTZ1-2* | ctatgaccaggtgccagtga | ctatgaccaggtgccagtga |
| *ACTB* | AGTACCCCATTGAACACGGT | ATACATGGCTGGGGTGTTGA |
