# Supplementary material for: Five glutathione S-transferase isozymes played crucial role in the detoxification of aflatoxin B1 in chicken liver
Source: J Anim Sci Biotechnol. 2025 Apr 8;16:54. doi: 10.1186/s40104-025-01189-7 (PMC11977921; doi:10.1186/s40104-025-01189-7)
Supplement: Supplementary file 3 — Additional file 3: Fig. S1. Homologies analysis between 17 GST proteins of Gallus gallus and 12 GST proteins from Meleagris gallopavo, Rattus norvegicus, Mus musculus, Macaca fascicularis and Homo sapiens. [file 40104_2025_1189_MOESM3_ESM.docx]

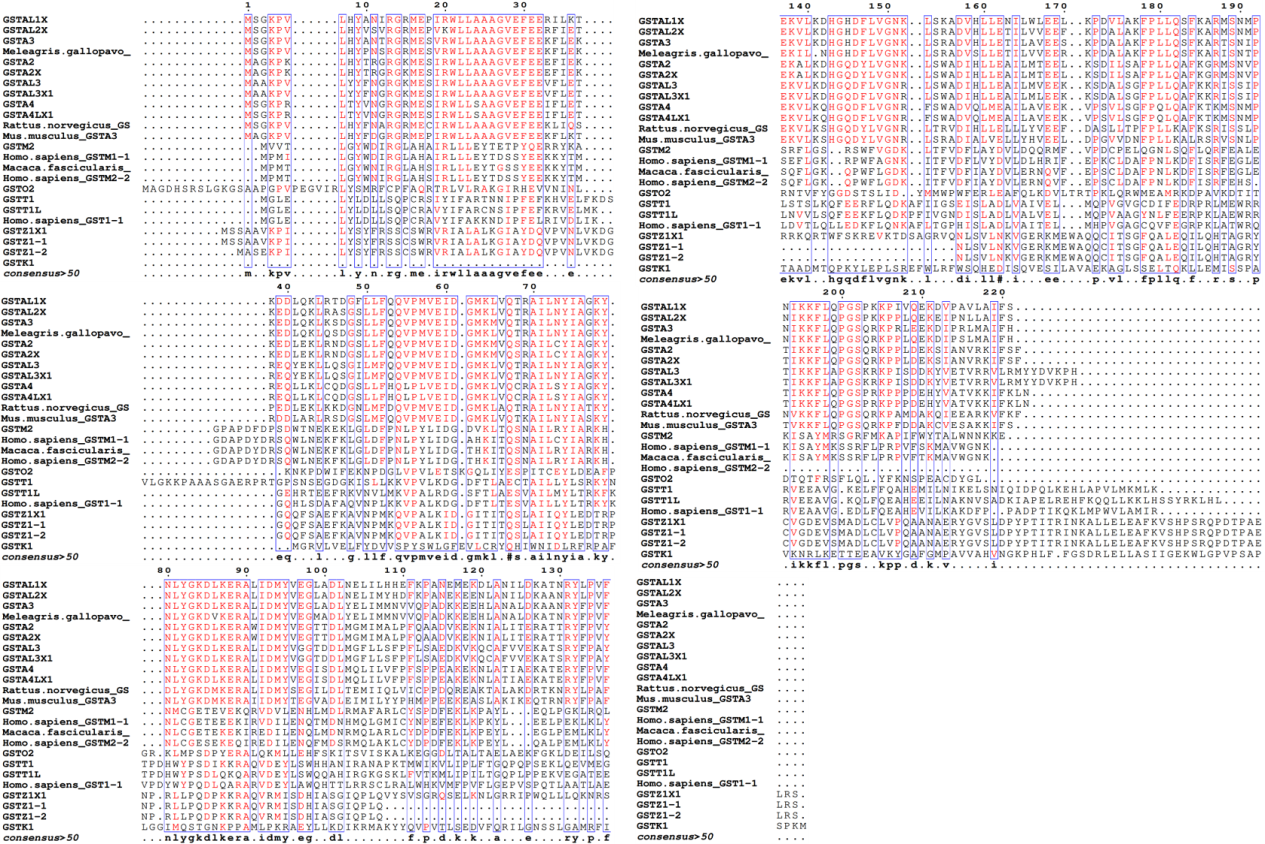


**Additional file 3: Fig. S1.** Homologies analysis between 17 GST proteins of *Gallus gallus* and 12 GST proteins from *Meleagris gallopavo*, *Rattus norvegicus*, *Mus musculus*, *Macaca fascicularis* and *Homo sapiens*
