# Supplementary material for: Five glutathione S-transferase isozymes played crucial role in the detoxification of aflatoxin B1 in chicken liver
Source: J Anim Sci Biotechnol. 2025 Apr 8;16:54. doi: 10.1186/s40104-025-01189-7 (PMC11977921; doi:10.1186/s40104-025-01189-7)
Supplement: Supplementary file 4 — Additional file 4: Table S3. The coverage and identity of 17 GST isozymes compared to GSTA3 of Mus musculus. [file 40104_2025_1189_MOESM4_ESM.docx]

**Additional file 4: Table S3.** The coverage and identity of 17 GST isozymes compared to GSTA3 of *Mus musculus*

| **Reference** | **Variety GTSs** | | **Coverage, %** | **Identity, %** |
| --- | --- | --- | --- | --- |
| GSTA3 *Mus musculus* | GSTA3 | *Mus musculus* | 100 | 100 |
| GSTA3 *Mus musculus* | GSTA5 | *Rattus norvegicus* | 99.55 | 84.09 |
| GSTA3 *Mus musculus* | GSTA1.2 | *Meleagris gallopavo* | 99.55 | 80.45 |
| GSTA3 *Mus musculus* | GSTAL1X | *Gallus gallus* | 100 | 79.64 |
| GSTA3 *Mus musculus* | GSTAL2X | *Gallus gallus* | 100 | 79.64 |
| GSTA3 *Mus musculus* | GSTA1.3 | *Meleagris gallopavo* | 100 | 79.19 |
| GSTA3 *Mus musculus* | GSTA1.1 | *Meleagris gallopavo* | 99.55 | 79.09 |
| GSTA3 *Mus musculus* | GSTA3 | *Gallus gallus* | 99.55 | 78.64 |
| GSTA3 *Mus musculus* | GSTA4LX1 | *Gallus gallus* | 99.55 | 78.18 |
| GSTA3 *Mus musculus* | GSTA4 | *Gallus gallus* | 99.55 | 78.18 |
| GSTA3 *Mus musculus* | GSTA2X | *Gallus gallus* | 100 | 77.83 |
| GSTA3 *Mus musculus* | GSTA2 | *Gallus gallus* | 100 | 77.83 |
| GSTA3 *Mus musculus* | GSTA2 | *Meleagris gallopavo* | 100 | 77.38 |
| GSTA3 *Mus musculus* | GSTA3 | *Meleagris gallopavo* | 99.55 | 77.27 |
| GSTA3 *Mus musculus* | GSTAL3 | *Gallus gallus* | 99.55 | 76.36 |
| GSTA3 *Mus musculus* | GSTA4 | *Meleagris gallopavo* | 99.55 | 76.36 |
| GSTA3 *Mus musculus* | GSTAL3X1 | *Gallus gallus* | 99.55 | 76.36 |
| GSTA3 *Mus musculus* | GSTZ1X1 | *Gallus gallus* | 45.25 | 46.02 |
| GSTA3 *Mus musculus* | GSTT1 | *Gallus gallus* | 79.64 | 44.85 |
| GSTA3 *Mus musculus* | GSTM2-2 | *Meleagris gallopavo* | 89.91 | 43.48 |
| GSTA3 *Mus musculus* | GSTZ1-1 | *Gallus gallus* | 82.11 | 43.08 |
| GSTA3 *Mus musculus* | GSTZ1-2 | *Gallus gallus* | 81.94 | 43.01 |
| GSTA3 *Mus musculus* | GSTM2-2 | *Homo sapiens* | 97.90 | 42.93 |
| GSTA3 *Mus musculus* | GSTM1-1 | *Homo sapiens* | 89.91 | 42.86 |
| GSTA3 *Mus musculus* | GSTM2 | *Gallus gallus* | 89.59 | 41.75 |
| GSTA3 *Mus musculus* | GSTT1L | *Gallus gallus* | < 30.00 | < 30.00 |
| GSTA3 *Mus musculus* | GSTT1-1 | *Homo sapiens* | < 30.00 | < 30.00 |
| GSTA3 *Mus musculus* | GSTK1 | *Gallus gallus* | < 30.00 | < 30.00 |
| GSTA3 *Mus musculus* | GSTO2 | *Gallus gallus* | < 30.00 | < 30.00 |
