# Supplementary material for: Five glutathione S-transferase isozymes played crucial role in the detoxification of aflatoxin B1 in chicken liver
Source: J Anim Sci Biotechnol. 2025 Apr 8;16:54. doi: 10.1186/s40104-025-01189-7 (PMC11977921; doi:10.1186/s40104-025-01189-7)
Supplement: Supplementary file 5 — Additional file 5: Fig. S2. The construction of pPICZαA-GSTA2X vector (A) and selection of positive single clones (B). [file 40104_2025_1189_MOESM5_ESM.docx]

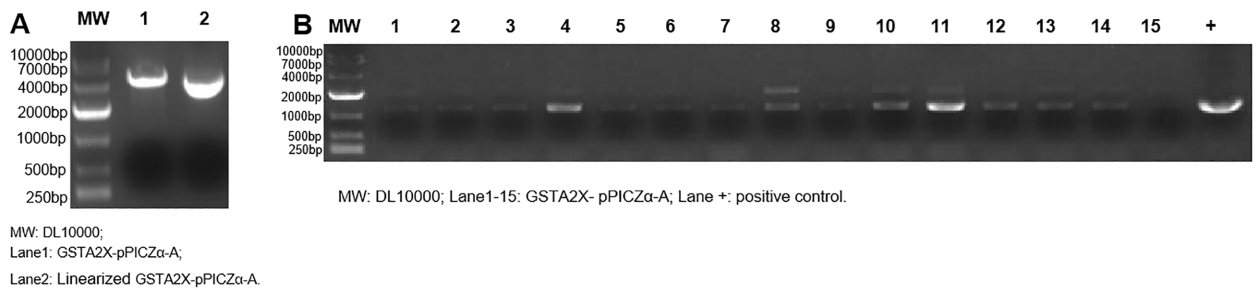


**Additional file 5: Fig. S2.** The construction of pPICZαA-GSTA2X vector (A) and selection of positive single clones (B).
